# Escalating Threat of Wheat Stripe Rust Under Climate Change: Pathogen Evolution, Resistance Durability, and Future Management

**DOI:** 10.3390/plants15071073

**Published:** 2026-03-31

**Authors:** Ameer Hamza Aslam, Zulfiqar Ali, Kamran Saleem, Rizwana Maqbool, Abdelfattah A. Dababat, Fatih Özdemir, Rachid Lahlali, Aziz Nurbekov, Moussa El Jarroudi, Sridhar Bhavani, Muhammad Amjad Ali

**Affiliations:** 1 Department of Plant Pathology, University of Agriculture Faisalabad, Faisalabad 38040, Pakistan; 2019ag7539@uaf.edu.pk; 2 Department of Plant Breeding and Genetics, University of Agriculture Faisalabad, Faisalabad 38040, Pakistan; zulfiqar_ali@uaf.edu.pk (Z.A.); rizwana.maqbool@uaf.edu.pk (R.M.); 3 Plant Protection Division, Nuclear Institute for Agriculture and Biology (NIAB), Faisalabad 38950, Pakistan; kamranniab@gmail.com; 4 International Maize and Wheat Improvement Center (CIMMYT), Cem Erserver Caddesi No: 9-11 Yenimahalle, 06810 Ankara, Türkiye; a.dababat@cgiar.org; 5 School of Agriculture, University of Jordan, Amman 11942, Jordan; 6 General Directorate of Agricultural Research and Policies (TAGEM), 06800 Ankara, Türkiye; ozdemirfatih@tarimorman.gov.tr; 7 Phytopathology Unit, Department of Plant and Environment Protection, École Nationale d’Agriculture de Meknès, km. 10, Route Haj Kaddour, B.P. S/40, Meknès 50001, Morocco; rlahlali@enameknes.ac.ma; 8 Plant Breeding and Oil Crop Department, Tashkent State Agrarian University, Tashkent 100140, Uzbekistan; azizbekisrail@gmail.com; 9 SPHERES Research Unit, Department of Environmental Sciences and Management, University of Liège, 6700 Arlon, Belgium; 10 Global Wheat Program, International Maize and Wheat Improvement Center (CIMMYT), Texcoco 56237, Mexico; sridharbhavani@outlook.com

**Keywords:** stripe rust, *Puccinia striiformis* f. sp. *tritici*, *Pst* biology, climate change, wheat

## Abstract

Stripe rust of wheat, caused by *Puccinia striiformis* f. sp. *tritici* (*Pst*), is one of the most devastating diseases that seriously threatens global wheat security. In the 21st century, *Pst* biology, epidemiology, and evolutionary pace have been altered far more quickly than expected because of climate variability. Warmer winters, along with erratic rainfall and increasing periods of leaf wetness, are continuously changing the geographic distribution of *Pst*. This may accelerate the emergence of races adapted to high temperatures and enhanced virulence, enabling their expansion into new agroecosystems. Despite extensive breeding efforts, varietal resistance is increasingly short-lived under the pressure of rapidly evolving lineages of the pathogen. *Pst* infection can be managed through integrative management practices, including biological control agents (BCAs), cultural and agronomic practices, rotation, and targeted application of fungicides. Varietal resistance, as well as disease management, is discussed in addition to recent advances in understanding pathogen biology, climatic influences, virulence evolution, and host resistance. Furthermore, this review highlights the need for climate-smart disease-resistant varieties breeding, a disease surveillance network, and diversified, eco-friendly control strategies to safeguard wheat production in an era of rapid environmental change.

## 1. Introduction

Wheat (*Triticum aestivum* L.) is considered a cornerstone of global food security by providing nearly one-fifth of the world’s caloric intake [1]. *Puccinia striiformis* f. sp. *tritici* (*Pst*), a pathogen responsible for causing stripe (yellow) rust disease of wheat, is among the most devastating foliar pathogens of wheat [2,3]. *Pst* infection reduces the photosynthetic area of infected leaves, resulting in high yield losses (ranging from 3% to 80%, depending on genotype and prevailing environmental conditions) [4]. Furthermore, it also deteriorates the quantity and quality of grains. Several disease epidemics were documented in Middle Eastern and Mediterranean countries from 2009 to 2010, causing severe yield losses. At the global scale, earlier estimates suggest that *Pst* can cause annual wheat yield losses of approximately 5.47 million tonnes across worldwide wheat-growing regions [5]. However, these estimates are largely derived from earlier assessments and may not fully capture the current dynamics of stripe rust under rapidly changing climatic conditions and evolving pathogen populations. More recent evidence indicates that disease impact is highly variable across regions and years, with localized epidemics frequently causing yield losses exceeding 50% in susceptible cultivars and, under highly favorable conditions, reaching up to 64–100% [6,7,8]. These observations suggest that global loss estimates should be interpreted with caution, as the economic and production impacts of stripe rust are increasingly driven by climate variability, regional epidemic intensity, and the emergence of more aggressive pathogen lineages. Beyond these economic impacts, the actual danger of *Pst* lies in its high genetic diversity, rapid turnover, and the ability of its spores (urediniospores) to spread over long distances, which facilitates its persistence and rapid spread across diverse agro-ecological zones [9].

Climate change has further complicated this scenario by reshaping pathogen behavior, plant physiology, and epidemiology of plant disease [10,11]. According to the IPCC Sixth Assessment Report (AR6, 2023), the world temperature will rise by 1.5 °C by the early 2030s [12]. Consequently, notable alterations in precipitation regimes, CO_2_ concentrations, and relative humidity will occur [13]. These changes will not only affect the microclimate within the crop canopies but also the epidemiology of many plant diseases, including stripe rust [14,15,16,17]. In parallel with changing climate conditions, breeding programs continually introduce new varieties to sustain wheat production under shifting environments [18]. Yet, *Pst* consistently evolves into new races, making it more challenging to control [19].

*Pst* is highly sensitive to temperature and moisture, and even small climatic shifts can influence its latent period, infection efficiency, sporulation rate, and survival [14,18,20]. Historically, stripe rust was regarded as a disease of cooler and moist temperate regions; however, since 2000, it has appeared in warmer and more arid zones. For instance, recent shifts in the geographic distribution of *Pst*, especially in North America, have been associated with temperature-specific adaptation [21]. This adaptation highlights the capacity of *Pst* populations to respond rapidly to environmental pressures, reinforcing the role of climate-driven selection in shaping pathogen evolution. Importantly, these climate-driven changes not only influence pathogen biology but also directly affect the durability of host resistance and the effectiveness of disease management strategies. The shifting dynamics of plant–pathogen interactions under changing climate conditions, the breakdown of varietal resistance within a few seasons, and the intensified use of fungicides together make stripe rust management an increasingly formidable challenge. These observations indicate the acquisition of thermal tolerance and an expanded ecological amplitude of *Pst* under changing climatic conditions [4,22,23]. In addition, the changing climate drivers (temperature, CO_2_, RH) may also alter defense signaling pathways, reduce the stability and effectiveness of resistance loci (*Yr* genes), and phenology of the host plant. In the context of all aforementioned issues, the effects of climate change on epidemiological processes across cellular, field, and regional scales remain insufficiently understood. Most previous reviews focus on the genetics of *Pst* [24,25,26], but few reports on how climatic range affects disease dynamics have been documented.

This review aims to bridge these disciplinary gaps by synthesizing current knowledge of stripe rust under a changing climate within an integrated conceptual framework. Specifically, it links climate-driven environmental variability with pathogen evolutionary dynamics and the durability of host resistance. Rather than treating temperature, moisture, host resistance, and pathogen biology as independent components, this review integrates these factors to explain how climate change influences *Pst* population structure, virulence evolution, and the breakdown of resistance. Furthermore, this review explicitly connects climate-driven pathogen adaptation with resistance breeding strategies and integrated disease management approaches, providing a unified perspective rather than treating these components separately. The following sections, therefore, examine the biology of *Pst*, its genetic variability and virulence patterns, host vulnerability, and the broader environmental drivers that collectively influence disease epidemics. Furthermore, we discuss mitigation strategies, including durable resistance breeding, BCAs, and advanced digital surveillance systems based on remote sensing and AI. This approach provides a holistic synthesis of multi-scale epidemiological processes and advanced management strategies, linking climate dynamics with pathogen evolution and practical disease control in the context of global food security.

## 2. Biology and Life Cycle of *Pst*

**Figure 1:**
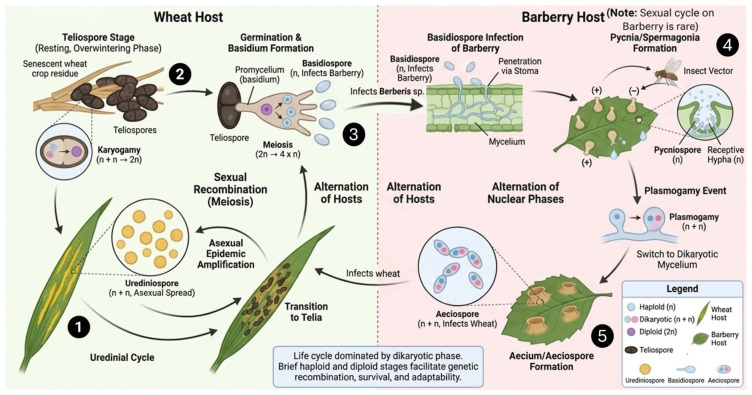
Biphasic life cycle of *Puccinia striiformis* f. sp. *tritici* showing alternation of hosts and nuclear phases. The life cycle is divided into five major stages based on spore types and host association. (1) Urediniospore stage (wheat host): Urediniospores (n + n) undergo rapid asexual multiplication on wheat, driving epidemic development. (2) Teliospore stage (wheat host): Toward the end of the season, black telia form containing teliospores, where karyogamy occurs (n + n → 2n), enabling overwintering. (3) Basidiospore stage (transition from wheat to alternate host): Teliospores germinate to produce basidia, where meiosis (2n → n) generates haploid basidiospores that infect the alternate host (*Berberis* spp.). (4) Pycnial stage (barberry host): Basidiospores form haploid mycelium and pycnia of opposite mating types; fertilization occurs via spermatia transfer (plasmogamy), restoring the dikaryotic state. (5) Aeciospore stage (barberry to wheat): Dikaryotic aecial hyphae produce aeciospores (n + n), which infect wheat and reinitiate the uredinial cycle. The life cycle is predominantly dikaryotic (n + n), with brief haploid and diploid phases facilitating genetic recombination. Sexual reproduction on barberry, although geographically limited in many wheat-growing regions, contributes to the emergence of new virulent races.

*Pst* is an obligate biotrophic fungus that belongs to the family *Pucciniaceae* and order *Pucciniales* [27]. It strictly depends on its host plant’s photosynthetic machinery for nutrition. The pathogen is heteroecious and completes its life cycle on two taxonomically distinct hosts and five spore stages, including pycniospores, aeciospores, urediniospores, teliospores, and basidiospores (Figure 1) [28,29]. Of the five spore stages of *Pst*, the uredinial and telial stages occur on the primary host (wheat). Teliospores germinate to produce basidia and basidiospores, which disperse and infect the alternate host (*Berberis* spp.), where the pycnial and aecial stages develop. The infection cycle of *Pst* is highly specialized, which involves several key steps: attachment of the spore to the leaf surface, germination, formation of an appressorium, penetration through stomata, and establishment of haustoria within a living mesophyll cell [2]. These haustoria are enclosed by a unique structure known as the extrahaustorial membrane, surrounded by a gel-like extrahaustorial matrix within living host cells (Figure 1). Urediniospores generally germinate under conditions of free moisture, with an optimum of 7–12 °C and an upper limit of about 20–26 °C. The urediniospores and teliospores are dikaryotic, while teliospores give rise to haploid basidiospores upon germination [30]. As nutrient depletion occurs in infected leaf tissues, the pathogen shifts from urediniospore production to the formation of teliospores (thick-walled spores that survive in adverse environments) [6]. This transition is typically associated with late stages of infection and is influenced by both environmental and host-related factors. Relatively lower temperatures, reduced nutrient availability, and changing moisture conditions can promote the differentiation of teliospores. In addition, host tissue senescence plays a critical role by altering physiological conditions within the leaf, thereby favoring the development of survival structures over asexual reproduction. During the following spring, under favorable conditions, teliospores germinate to produce basidia and basidiospores, which infect alternate hosts (such as *Berberis chinensis*, *B. holstii*, *B. koreana*, and *B. vulgaris*), where pycnial and aecial stages are initiated [31]. *Pst* is known to possess a functional sexual cycle involving *Berberis* spp. as alternate hosts, where pycnial and aecial stages are produced. However, its epidemiological significance in field populations appears to be regionally constrained. Although sexual recombination can generate novel multilocus genotypes under experimental and localized natural conditions, its epidemiological contribution is limited but regionally important, with most global populations dominated by clonal lineages. In most wheat-growing regions, evolutionary dynamics are primarily driven by stepwise mutations, somatic recombination, gene duplication, and long-distance dispersal of asexual urediniospores rather than frequent sexual recombination [32]. Therefore, unlike *Puccinia graminis* f. sp. *tritici*, where sexual reproduction plays a central epidemiological role, the contribution of the sexual cycle in *Pst* is considered limited and highly context dependent. Figure 1 depicts the complete life cycle of *Pst*.

However, the overwintering biology of *Pst* varies across geographic regions and climatic conditions. In cooler temperate regions, such as parts of Europe and North America, the pathogen primarily survives as mycelium in infected winter wheat or volunteer plants, which serve as a source of inoculum for early-season infections [2]. In contrast, in regions with harsh winters or in the absence of suitable hosts, long-distance dispersal of urediniospores from distant source regions plays a major role in epidemic initiation [33]. In areas where *Berberis* spp. occur, sexual recombination can contribute to local genetic diversity; however, its contribution to disease epidemiology is generally limited and varies among regions [31]. In relatively warmer regions, particularly in parts of South Asia and in highland areas of Africa, mild winter conditions and the continuous presence of host plants can facilitate pathogen survival and repeated infection cycles, contributing to sustained disease pressure [34,35,36]. These geographic differences highlight the importance of regional climate and host availability in shaping overwintering strategies and epidemic development of *Pst*.

## 3. Symptoms and Disease Development of Stripe Rust

**Figure 2:**
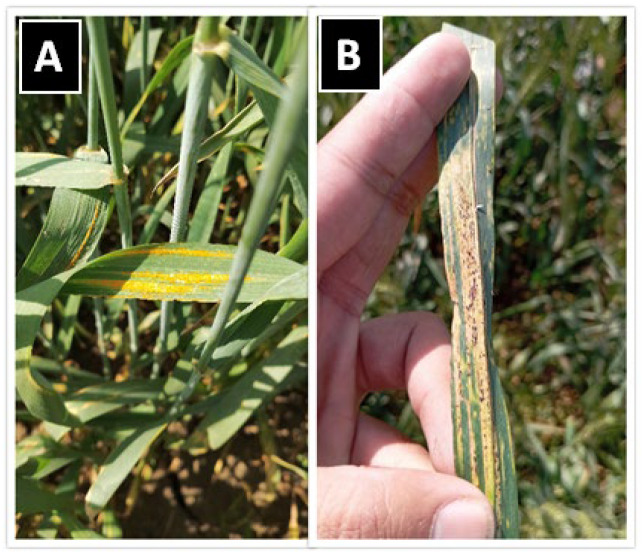
Symptoms of stripe rust caused by *Puccinia striiformis* f. sp. *tritici* on wheat. (**A**) Early-stage infection showing chlorotic stripes with developing uredinial pustules. (**B**) Advanced infection stage with well-developed yellow-orange uredinia arranged in linear streaks along leaf veins. These symptoms reduce photosynthetic area and contribute to yield loss.

The first apparent symptoms (small spots, chlorotic flecks, and pustules) of *Pst* are usually observed after 7 to 14 days (under conducive conditions) of spore landing on the leaf surface of susceptible wheat varieties; however, the symptoms are limited to small spots and chlorotic flecks on the leaves of resistant varieties. Upon the progression of infection, these spots develop into elongated yellow-orange pustules forming longitudinal stripes on leaves (as shown in Figure 2A), sheaths, glumes, and awns [37,38]. After pustule maturation, uredinia rupture and release masses of yellow-orange urediniospores. As the pathogen depletes nutrients and water with time, accompanied by the rise in temperature, the infected tissues turn dark brown and dry, reducing photosynthetic capacity and plant vigor and causing leaf desiccation [2]. In regions where the sexual cycle occurs, teliospores may form on wheat leaves under specific conditions. Teliospores are dark brown to black (Figure 2B), thick-walled, two-celled spores that function primarily as survival structures. These teliospores form basidia upon germination that are capable of infecting the *Berberis* spp. [25,27,35,36,39]. Typical symptoms of stripe rust include yellow-orange uredinial pustules arranged in linear stripes on leaf surfaces (Figure 2A,B).

## 4. Global Epidemiology of Wheat Stripe Rust

**Figure 3:**
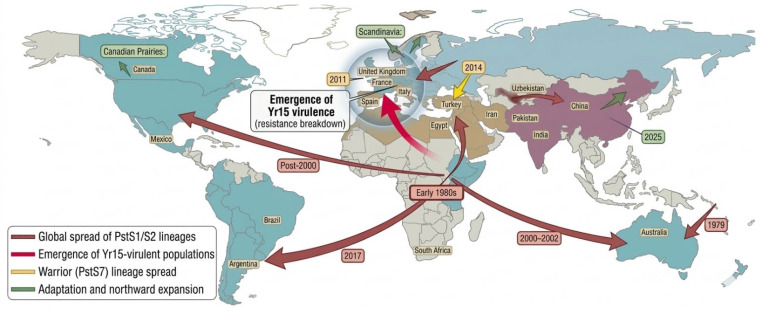
The map illustrates the origins and migration routes of the most significant *Pst* lineages worldwide. Red arrows represent the primary global spread of the *Pst*S1 and *Pst*S2 lineages. A small red arrow pointing into Australia marks the historical first detection of *Pst* there in 1979. One prominent red arrow stretches westward from the Middle East and East Africa across the Atlantic Ocean, showing the dispersal of new lineages (*PstS1* (East African origin) and *PstS2* (Middle East/Central Asia) to the Americas after the year 2000. A second long red arrow arcs southeastward from the same region toward Australia, indicating a similar dispersal event where these new lineages replaced older pathogen populations between 2000 and 2002. In Asia, a shorter red arrow points east, visualizing the gene flow of *Pst* moving from Uzbekistan into Xinjiang, China. The yellow arrows focus on the spread of the aggressive Warrior race (*PstS7*). A specific yellow arrow points southeast from Europe into Turkey, marking a critical event in 2014 where the Warrior race (*PstS7*) entered the region. This movement is significant because the map notes that wheat cultivars in Turkey, which were previously resistant to the fungus, became susceptible upon the arrival of this new race. Small green arrows in the Canadian Prairies and Scandinavia (UK) point northward, visually representing the pathogen expansion into colder, northern latitudes. A study reported the phenotypic and molecular characterization of *Pst* isolates collected in Uruguay and Argentina in 2017, highlighting the emergence and spread of new virulent lineages in South America. In contrast, the emergence of *Yr15*-virulent races has been documented in central and western Europe, indicating the recent breakdown of *Yr15* resistance under field conditions. These patterns are illustrated in Figure 3 using directional arrows to indicate the geographic spread of virulent lineages across different regions. Recent studies revealed adaptation of *Pst* to heat, noting that in China, specific races (*CYR32*, *CYR33*, and *CYR34*) have evolved to survive and reproduce at temperatures up to 27 °C. The data used to generate the map were acquired from [23,42,60,62,63,64,70,71,74,75,76,77].

The distribution and intensity of *Pst* have changed dramatically over recent decades, largely driven by shifts in climatic patterns and the emergence of new, thermotolerant races (Figure 3). Increasingly variable temperatures, irregular rainfall patterns, and extended periods of humidity have created favorable conditions for pathogen survival, dispersal, and aggressiveness. As a result, stripe rust epidemics are now emerging in regions that were previously considered climatically unsuitable [40]. Earlier global assessments by Beddow, Pardey, Chai, Hurley, Kriticos, Braun, Park, Cuddy and Yonow [5] estimated that the annual global wheat yield losses caused by *Pst* amount to nearly 5.47 million tonnes, equivalent to USD 979 million per year. However, more recent studies indicate that the impact of stripe rust is highly dynamic and influenced by changing climatic conditions, the emergence of thermotolerant and aggressive lineages, and regional variability in host susceptibility [8,40]. In severe epidemic years, the global losses due to wheat rust diseases have surpassed USD 3 billion [41].

These outbreaks, though geographically distinct, reveal clear global patterns in stripe rust epidemiology. Climate variability has expanded *Pst* into new regions. At the same time, the spread of aggressive, thermotolerant lineages (*PstS1*, *PstS2*, Warrior types) reflect strong global connectivity via wind and human activity. Earlier infection onset and longer disease periods further indicate enhanced pathogen fitness. These observations suggest that stripe rust epidemics are no longer isolated regional events but components of a globally interconnected and climate-sensitive pathosystem.

A major cause of these losses is the rapid emergence and spread of thermotolerant and virulent lineages such as *PstS1* and *PstS2* (Figure 3). These races, first identified in East Africa, have demonstrated remarkable adaptability to warmer environments [29,42]. In 2010, the disease spread rapidly and infected 600,000 hectares with yield losses of nearly 50% [43]. A review report also stated that yield losses caused by *Pst* ranged from 2.7% to 96.7%, depending on the degree of susceptibility of the genotype [44]. These global drivers are consistently reflected across major wheat-growing regions.

In North America, for example, epidemic dynamics illustrate the interaction between climatic variability and pathogen adaptation. Between 2000 and 2007, the disease was reported annually in more than 15 U.S. states and resulted in estimated yield losses exceeding 6.5 million tonnes [29]. It resulted in an additional 2.2 million tonnes of yield loss and about USD 30 million spent on fungicide applications in Washington State alone [45]. These outbreaks were strongly modulated by environmental conditions, where drought temporarily suppressed disease in the Great Plains, while mild and wet conditions in the Pacific Northwest triggered severe epidemics. Yield reductions reached up to 57.5% (susceptible winter variety) and 35.9% (susceptible spring wheat) during 2012. Similarly, Brar [46] documents that western provinces of Canada experienced widespread epidemics during 2010–2011, which caused significant losses.

A similar climate-driven and connectivity-linked pattern is evident in South America, where stripe rust has shifted from sporadic occurrence to a persistent regional threat. After nearly eight decades of limited occurrence, devastating epidemics struck Uruguay and Argentina in 2017 following a period of unusually cool and wet conditions [47,48]. As recorded by Carmona, et al. [49] and Carmona et al. [47], Argentina experienced the worst epidemics since the 1930s, with approximately 3,000,000 hectares affected in 2017. These outbreaks illustrate how shifting climatic regimes can drive the pathogen populations into a more aggressive form that can lead to large-scale disease resurgence.

In Europe, long-term epidemic patterns further demonstrate how warming trends and changing seasonal dynamics enhance pathogen survival and spread. The Warrior and Kranich lineages, which emerged in 2009–2011, spread rapidly across the UK, France, Germany, Denmark, and northern Europe [50]. Warmer winters with longer wet springs have facilitated the overwintering and early-season infection of *Pst* populations [51]. The resurgence of the pathogen in Portugal after two decades of absence highlights that a climatic moderation in southern Europe is reshaping the disease landscape [52]. In Serbia, national and experimental reports revealed that the rising spring temperatures and increased humidity since 2014 have driven stripe rust to dominate over leaf rust, with recorded disease severity reaching up to 90% in experimental plots [51]. Australia, despite its proactive national breeding and monitoring programs, continues to experience periodic epidemics of yellow rust exacerbated by mild winters and extended dew periods. The annual cost of fungicide applications between 2003 and 2006 was between USD 40–90 million [53]. The 2010 epidemic in Ethiopia, fueled by extended cool and humid conditions, devastated commercial wheat fields. This epidemic led to yield losses of up to 100% in susceptible varieties, and more than USD 3 million was spent on fungicide [54].

In South Asia and China, these globally consistent drivers are particularly evident under high cropping intensity and favorable climatic conditions. In India, recurring epidemics have become common in the cooler northern regions. Studies reported that stripe rust affected 10 million hectares of wheat [55]. Punjab faced severe epidemics in the years 2008 and again during 2010–2011, favored by prolonged dew periods and mild temperatures. This disease resulted in losses of 236 crore INR [56]. In Pakistan, similar climate-favored outbreaks were observed in 2005 and 2010, particularly in irrigated and rainfed zonesm [57]. The spread of thermotolerant races, for example, *PstS1* and *PstS2*, along with warmer winters and increased humidity, has made stripe rust a recurring and more damaging disease across the Indo-Gangetic plains. In China, a severe outbreak in 2002 affected over 6.6 million hectares across 11 provinces [58], and up to 13.8 million tonnes of yield losses were reported by Zhao and Kang [59]. Subsequent monitoring indicates that warmer winters now allow the pathogen to persist and migrate earlier in the growing season. These conditions, coupled with the emergence of high-temperature-adapted races, have transformed stripe rust into a year-round challenge for Chinese wheat systems. These region-specific observations collectively highlight a high degree of global connectivity in *Pst* populations, in which emerging lineages rapidly transcend geographical boundaries and establish themselves across diverse agro-climatic zones. Large-scale surveillance and genotyping efforts coordinated through the Global Rust Reference Center (GRRC) have documented how a limited set of highly divergent and globally distributed *Pst* lineages, including *PstS1*, *PstS2*, *PstS7*, *PstS8*, and *PstS10*, are responsible for major epidemics across the continents. Long-term GRRC monitoring has revealed that some of these lineages have rapidly displaced older, locally adapted populations. This coordinated global evidence strongly supports the concept of a dynamically interconnected pathogen system shaped by both climate and long-distance dispersal [60].

The global patterns of stripe rust epidemics are primarily shaped by a combination of clonal evolution, mutation-driven diversification, and long-distance dispersal rather than frequent sexual recombination. Although sexual recombination on *Berberis* spp. can generate novel genetic combinations, its contribution to natural field populations is generally limited and geographically constrained. In many wheat-growing regions, where alternate hosts are absent or rare, populations of *Pst* are predominantly clonal. In addition, Lei, et al. [61] have reported through experimental evidence that somatic hybridization and mitotic recombination between co-infecting strains can generate new virulence phenotypes in the absence of sexual reproduction. Furthermore, mutation-driven evolution, including point mutations and gene duplication, acts in combination with somatic recombination to accelerate pathogen adaptation under changing climates. Recent reports provide evidence of the emergence and migration of new *Pst* lineages (Table 1), closely associated with changing climatic conditions, across Europe and Asia but also into other continents. These observations suggest that future *Pst* populations will likely exhibit increased thermal adaptation, broader virulence spectra, and enhanced epidemic potential under changing climatic conditions.

**Table 1:** Documented cases of geographic expansion, lineage diversification, and epidemiological shifts of *Pst* in relation to recent climatic changes.

Events	Observed Changes and Expansion in the Pathogen	Associated Climatic Factors	Impact on Disease Dynamics and Host Range	References
Australia (1979–present)	First detection and adaptation of *Pst* to warmer, drier southern hemisphere environments.	Initial introduction from Europe Evolved thermal tolerance for local climates.	Persistent epidemics established despite initial climatic unsuitability.	[62,63,64]
Syria and Lebanon (1994–1999)	Emergence of virulence factors v2-v9 that later characterized *PstS2*	Mild winters and periodic rainfall	Early evidence of the pre-emergence stage of modern high-temperature-adapted lineages.	[65,66]
East Africa → Middle East → Global (2000–2002)	Emergence and global dispersal of *PstS1* (East African origin) and *PstS2* (Middle East/Central Asia). These lineages spread to the Americas and Australia, replacing older populations.	Adaptation to higher-temperature regimes and increased urediniospore production, favored by warm, dry conditions.	Increased aggressiveness, broader virulence spectrum, and expansion into previously unsuitable warmer regions.	[67,68]
Eastern Mediterranean (2005–2006)	Characterization of 12 distinct *Pst* pathotypes, including *PstS2-v27*	Warm, dry summers and variable winters promoted survival and recombination.	Identified the region as a hotspot for new *Pst* variants and a bridge zone between African, Asian, and European populations.	[40,69]
North Africa and West Asia (2010–present)	Emergence of *PstS1*/*PstS2-v1*, *v3* and *v27* variants gained virulence against *Yr1*, *Yr3*, *Yr10*, *Yr27*.	Warming springs and extended leaf wetness periods favor high urediniospore loads	Breakdown of widely deployed *Yr* genes Establishment of heat-tolerant varieties	[60,65]
Europe (2011–present)	Emergence of Warrior (*PstS7*) and Kranich (*PstS8*) lineages. Replacement of old European populations	Milder winters and prolonged wet periods favored survival and infection cycles.	Rapid lineage turnover and increased virulence in temperate Europe.	[66,67,68]
Turkey (incursion of Warrior race, 2014)	First confirmed detection of Warrior (*PstS7*) race in Turkey in 2014. Previously resistant Turkish cultivars became susceptible.	Arrival of thermotolerant lineage in a region prone to warm springs and early epidemics.	New lineage found in Turkey, indicating cross-region movement and breakdown of local resistance.	[64]
Northward expansion (Europe and North America)	Increasing *Pst* occurrence in Scandinavia, the UK highlands, and Canadian prairies.	Warmer winters permit overwintering at higher latitudes.	Poleward movement of viable pathogen populations.	[60,70,71]
East Africa and the Middle East	High-temperature-adapted *Pst* populations originating in these regions.	Seasonal migration is linked with monsoon and temperature shifts.	Source of globally dispersed, heat-tolerant races.	[65,72]
Central Asia → Xinjiang, China	Gene flow of *Pst* lineages from Uzbekistan to the Xinjiang region.	Regional climatic connectivity and wind-borne dispersal across arid zones.	Demonstrated cross-border migration of clonal lineages.	[73]
China (2025)	Races *CYR32*, *CYR33*, and *CYR34* survived and reproduced at temperatures up to 27 °C.	Rising mean temperatures expanded oversummering zones northward.	Heat adaptation reshaped regional epidemiology and overwintering dynamics.	[23]
Global (2009–2015)	Dominance of a few highly divergent lineages (*PstS1*, *PstS2*, *PstS7*, *PstS8*, *PstS10*)	Cross-continental dispersal enhanced by wind trajectories and climate connectivity.	Aggressive races caused simultaneous epidemics across Asia, Europe, and Africa.	[60]

## 5. Climate Change Impacts on Wheat Yield and Global *Pst* Dynamics

Climate change is reshaping the epidemiology of *Pst* through multiple interacting environmental drivers, including temperature, moisture, atmospheric CO_2_ concentration, and the increasing frequency of extreme climatic events. These factors do not act independently but collectively influence pathogen development, host susceptibility, and disease dynamics across spatial and temporal scales. In this section, the major climatic determinants of stripe rust epidemics are synthesized to provide a unified framework linking environmental change with pathogen evolution and disease risk.

### 5.1. Temperature Effects on Pst Development and Virulence

Temperature is a primary driver of *Pst* biology, epidemiology, and geographic distribution. *Pst* was historically considered a cool-season pathogen, as urediniospores germinate and successfully infect at 2–15 °C, with classical optimal conditions 7–12 °C under high RH (60–70%). Infection declines above 15–20 °C in many isolates (infection success rate falls from 100% at 15 °C to nearly 1–5% at 20–24 °C) [2,78,79]. A classical study reported that a temperature range from 0 to 23 °C is the favorable temperature for disease development, with minimal activity at 0 °C, optimal progress at 11–12 °C, and inhibition occurring at 23–24 °C [80]. Cooler (0–13 °C) and humid nights (RH > 70%) favor the disease development and progression [2]. For example, Hailu and Fininsa [81] and Hundie, et al. [82] reported the mean minimum (7.9 °C) and maximum (21 °C) temperatures during the main season at the Agrfa and Sinana regions of Africa fall within the optimal window for stripe rust epidemic development. Recent shifts in climate change have substantially affected the stripe rust as compared to other rusts [83].

However, recent climate-driven shifts in global temperature have profoundly impacted the thermal adaptation of *Pst* populations. Over the past decade, population genomic, phenotypic, and epidemiological studies reported the emergence and spread of *Pst* populations that can severely infect wheat at warmer temperatures, showing improved performance across approximately 12–28 °C, with optimal activity around 18–25 °C, have been increasingly reported [68,84]. Novel, highly aggressive, and thermotolerant lineages appeared after 2011. These warmer-adapted populations were called *PstS1*/*PstS2*/*PstS7*/*PstS10* and other related genotypes. Such lineages were later named Warrior. These races entered Europe and rapidly displaced many historical populations, bringing new virulence combinations having greater epidemic potential. Pathogenomic and population surveys show these introductions were rapid, widespread, and associated with marked increases in prevalence as Warrior/Kranich-type isolates rose to dominate many country-level samples within a few seasons [50,60,85]. This shift has expanded the thermal niche of *Pst*, enabling clear differentiation between classical cool-adapted populations and newly emerged thermotolerant lineages. These distinct thermal thresholds are summarized in Table 2 to differentiate classical and thermotolerant populations. Recent experiments, for example, demonstrated that some modern *Pst* races remain viable and capable of reproducing at mean temperatures up to 27 °C, revealing adaptation potential under warming conditions [23]. Thus, classical optimum temperatures (7–12 °C), thermotolerant optimum ranges (around 18–25 °C), and upper survival limits (25–27 °C) represent distinct biological thresholds of *Pst*, reflecting adaptation from cool-season specialization to expanded thermal tolerance under climate change.

Temperature also affects the overwintering, oversummering, and seasonal persistence of the pathogen [86,87]. In general, infection progresses slowly in cooler months (December and January) and more rapidly in moderately cooler months (February) [88]. Several findings revealed that *Pst* can survive under cold conditions even at 0 °C [89,90,91]. Although prolonged exposure to low temperatures reduces the viability of urediniospores, resulting in low or no disease onset [89,92]. Findings of Chen, et al. [93] revealed that field-based teliospore germinability from Qinghai ranged from 23.9% (January) to 4.4% (June) in 2018 and from 10.3% (January) to 6.0% (May) in 2019 (nearly 5.9%), which indicates the capacity for overwinter survival in cool climates. In contrast, high temperatures (>30 °C) decrease spore viability and infection success [34,94]. In the context of climate change, warm winters have expanded the geographic area where pathogens (such as *Pst*) can overwinter locally. This has significantly altered traditional refugia of disease development, thereby increasing the local inoculum reservoir and advancing the seasonal start of epidemics. Spread of high-temperature-adapted isolates resulted in shortened latent period of the pathogen [95]. Many studies have shown that reduced latent periods at elevated temperature compared to historical strains result in more infection cycles per season and faster epidemic build-up. Additionally, epidemiological studies revealed that latent periods vary greatly with temperature, typically 6–11 days at 15–20 °C but can extend to 118 days, or even beyond 188 days under snow cover in cold regions [96]. Temperature requirements for different developmental stages of *Pst* are given in Table 2. In addition to temperature, moisture availability plays a critical role in determining infection success and epidemic development.

**Table 2:** Temperature requirements for developmental stages of *Puccinia striiformis* f. sp. *tritici* (*Pst*): comparison between classical and thermotolerant populations.

Developmental Stage	Classical *Pst* (°C)	Thermotolerant *Pst* (°C)	References
Germination	0–2 (min), 9–13 (opt), 26 (max)	Germination sustained at higher temperatures (~15–20 °C)	[2,8]
Infection efficiency	7–12 (opt)	Effective infection in thermotolerant populations at ≥15–20 °C	[71,95,97]
Growth & colonization	12–17 (opt)	Maintained at elevated temperatures (15–22 °C, reduced efficiency)	[13]
Sporulation	13–18 (opt)	Sustained at higher temperatures (18–22 °C)	[31]
Upper thermal limit	inhibited > 23–24 °C	Active up to approximately 25–27 °C (upper survival limit, not optimal)	[2,13]

Whereas: ~ = approximately; ≥ = greater than or equal to; min = minimum temperature required for the initiation of a given developmental process; opt = optimal temperature range at which the process occurs most efficiently; max = maximum temperature at which the process can occur before inhibition. Temperature ranges for classical *Pst* represent optimal conditions for different developmental stages, whereas values reported for thermotolerant populations reflect expanded thermal tolerance, including the ability to maintain infection, growth, and limited sporulation at higher temperatures. Temperatures approaching 25–27 °C represent the upper limits of pathogen survival and limited reproduction rather than optimal conditions for epidemic development. Differences between classical and thermotolerant populations reflect adaptive responses of *Pst* under changing climatic conditions. Thermotolerant populations refer to recently evolved lineages (e.g., *PstS1*, *PstS2*, Warrior types) exhibiting expanded thermal adaptation.

### 5.2. Moisture and Humidity Effects on Infection Dynamics

RH and leaf wetness are critical microclimatic factors for the progression of *Pst* epidemics. Urediniospores of *Pst* require RH over 95% to initiate germination and a leaf wetness duration of at least 3 h [2]. Under optimal environmental conditions (7–12 °C, RH > 95%), urediniospores can germinate in less than 3 h, while continuous leaf wetness for 20 h can result in infection rates exceeding 80% [98].

Moisture requirements can differ depending on the stage of *Pst*. For example, infection of the alternate host (*Berberis*) requires longer leaf wetness duration. Ahmad et al. [57] noted that teliospore germination, basidiospore production, and germination and germ tube penetration require a minimum of 32 h of leaf wetness under the optimum temperature of around 10 °C. Similarly, Singh, et al. [99] stated that at lower temperatures, a longer duration of leaf wetness is needed; a dew period of about 12 h is required for infection establishment. Extended dew periods, frequent rainfall, or prolonged high nighttime humidity greatly increase the infection cycles of the pathogen within the season [100,101].

Climate change is significantly altering these moisture-related dynamics and reshaping the epidemiology of stripe rust. In temperate wheat growing areas, warmer nights and higher atmospheric water vapor content have increased the frequency of humid nights, promoting more frequent and longer dew periods. This creates an ideal microclimate for early-season *Pst* infections [100]. Modeling studies have predicted that with a 1 °C rise in global temperature, the atmosphere can hold around 7% more water vapor [12]. As a result, conditions that previously limited pathogen establishment, especially areas once too dry for *Pst* development, may become suitable for infection. Conversely, drier and hotter areas may experience opposite trends. In arid and semi-arid environments where higher daytime temperatures exceed 25 °C, lower humidity suppresses spores’ viability and germ tube formation [71]. These contrasting outcomes highlight how shifts in rainfall patterns and humidity under a warming climate influence the geographic expansion, distribution, timing, and severity of stripe rust epidemics. Beyond temperature and moisture, rising atmospheric CO_2_ levels further influence host–pathogen interactions.

### 5.3. Elevated CO_2_ and Host-Pathogen Interactions

Elevated atmospheric CO_2_ (eCO_2_) causes a series of host physiological changes that directly or indirectly shape *Pst* development [102]. One of the major effects of eCO_2_ is the reduction in stomatal conductance by approximately 34% and stomatal density by nearly 14.3% (SE ± 2.2%) per 100 ppm increase in CO_2_ concentration. This may influence stomatal-mediated infection processes [103,104,105].

Higher concentrations of CO_2_ generally increase photosynthetic rates and increase canopy growth, the leaf area index (LAI) by 10–40%, and total crop biomass by 7–27% (Table 3) [104,106,107]. As a result, larger and denser canopies extend the green leaf period, reduce within-canopy ventilation, and increase leaf wetness duration [108]. Such structural changes create microclimatic conditions favorable for *Pst* development. For instance, FACE (Free-Air CO_2_ Enrichment) studies have reported biomass increases of 10–30% and nocturnal humidity rises of 5–10% under eCO_2_ conditions, facilitating more frequent infection cycles in conducive environments [109,110]. Additionally, eCO_2_ also causes nutrient dilution. Higher carbon assimilation reduces leaf nitrogen concentrations by 10–15% and increases the C:N ratio in plant tissues, which can alter host resistance and pathogen performance. While low nitrogen can limit sporulation in some cases, it can also weaken host defense pathways that depend on nitrogen-rich metabolites [111,112]. In parallel with gradual climatic shifts, extreme weather events are increasingly shaping disease dynamics in unpredictable ways.

### 5.4. Extreme Climatic Events and Epidemiological Consequences

**Figure 4:**
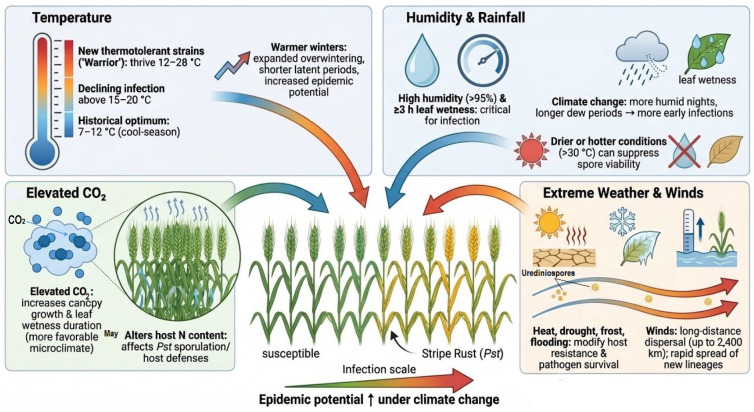
A pictorial representation of the influence of climatic and environmental variables on the epidemiology of stripe rust disease of wheat. ↑ shows an elevated temperature.

Extreme climatic events such as winds, heatwaves, frost, and drought have become more frequent and intense under the current global climate change. Sudden and often unpredictable extreme climatic events, such as heat waves, drought, and irregular precipitation patterns, can significantly influence the epidemiology of *Pst* by altering pathogen development and weakening host defense mechanisms (Table 3). Such events alter host-pathogen interactions by affecting spore survival and modifying the efficiency of host resistance mechanisms [113]. Winds play an important role in the aerial dispersal of urediniospores, transporting virulent *Pst* races across vast geographic regions (Figure 4). Atmospheric and field observations have proven that urediniospores can survive long enough to be transported hundreds to thousands of kilometers in upper air currents. Documented dispersal distances by urediniospores of *Pst* include 500 km [33], 800–1200 km [2], and up to 1700–2400 [114] km across continents. Moreover, heat waves and sudden temperature fluctuations can temporarily suppress the rust development. A study documented that stripe rust infection success falls from 100% at 15.4 °C to 0.8% at 20.5 °C in laboratory infection assays [23] while many new *Pst* lineages can tolerate up to 20–28 °C [115]. Heat stress disturbs antioxidant enzyme systems and hormonal crosstalk in the host plant, reducing the efficiency of salicylic acid-mediated resistance pathways [116]. These extreme climatic factors collectively influence pathogen development and disease progression (Table 3).

**Table 3:** Environmental factors alter the infection processes, dispersal patterns, survival capacity, and evolutionary potential of *Pst*.

Environmental Factor	Biological Responses and Disease Implications	Examples	References
Climate warming (↑ mean temp, ↑ extremes)	↓ Latent periods ↑ Aggressiveness of *Pst* ↑ Expansion into warmer and higher-latitude zones.	Reported thermotolerant and more aggressive strains Documented epidemics in warmer areas (U.S., Australia, China).	[60,68,71]
High relative humidity and leaf wetness	↑ Germination rate ↑ Infection success ↑ Number of infection cycles per season	RH and leaf wetness thresholds documented (RH: 92–95%, leaf wetness 3–8 h for infection)	[68,117,118]
Extreme weather (more frequent heatwaves, heavy rainfall, drought spells, freeze-thaw)	Weaken host defense (heat, drought, frost interactions) and ↑ Chances for epidemic spread	Numerous case studies linking extreme anomalies to major epidemics (Ethiopia 2010; North America 2010)	[42,60,119,120]
Increased frequency and intensity of storms and wind speed	↑ Potential for long-distance airborne transport ↑ Chance of intercontinental introductions of exotic lineages	Global lineage introductions (Warrior/Kranich) to new and previously unsuitable areas and documented long-distance dispersal patterns	[42,85,121]
eCO_2_	↑ LAI Altered N dilution Stomatal changes may reduce entry in some contexts	Mixed results from FACE and chamber studies for foliar pathogens Mechanisms applicable to *Pst* via canopy microclimate changes	[97,122,123]
Increased pathogen survival (overwintering and oversummering) due to milder winters or altered seasonality	Extended survival in regions previously marginal ↑ Baseline inoculum for early-season epidemics	*Pst* oversummering/oversummering region shifts Chinese *CYR* races show higher thermal tolerance Northern Europe overwintering reports	[23,60,64]
Genetic recombination, somatic hybridization, and high evolutionary potential	↑ Emergence of novel virulence combinations and possible host-range shifts Somatic hybridization is documented in rusts and linked to new virulence	Somatic hybridization/recombination documented in *Puccinia* and related rust genera; implicated in rapid virulence change	[85,117]

Whereas ↑ indicates an increase and ↓ indicates a decrease.

Frost and freeze–thaw cycles cause cellular damage and membrane leakage (reported up to almost 50%) after exposure to −4 °C for 10.5 h in plants [124]. Such damage results in susceptibility of the host plant. Low-temperature events may also reduce the viability of urediniospores of *Pst*. Study of Ma, et al. [89] showed that *Pst* mycelia inside green leaves were killed by low temperatures. Results of this study further revealed that the *Pst* infection largely disappeared at −10 °C, −15 °C, and −20 °C. Drought stress, followed by sudden moisture availability, further enhances infection potential by disrupting the redox balance and hormonal homeostasis in the plant. It specifically reduces salicylic acid signaling and promotes abscisic acid pathways that favor biotrophic pathogens [116,125]. On the other hand, extended rain and high humidity (>95%) generate optimal microclimates for spore germination and stomatal penetration, significantly accelerating epidemic development [126]. Flooding conditions reduce oxygen availability to roots and alter photosynthetic capacity, hence lowering host vigor and disease resistance [127,128]. Such stresses create temporal windows of susceptibility that allow rapid *Pst* colonization and disease progression.

## 6. Mechanisms of Resistance Breakdown in Wheat–*Pst* Interactions

Resistance breakdown in wheat–*Pst* interactions can be understood through the interplay of race-specific resistance, quantitative resistance (APR/HTAP), and their sensitivity to climatic and evolutionary pressures. The durability of wheat resistance to *Pst* is challenged by dual pressures of pathogen evolution and climate-mediated host vulnerability. For mechanistic clarity, resistance breakdown can be interpreted across three interconnected layers: (i) pathogen recognition, (ii) signal transduction, and (iii) downstream defense responses.

### 6.1. Recognition Layer: Yr Genes and Effector-Mediated Evasion

Race-specific (all-stage) resistance (ASR) operates primarily at the recognition level and is governed by *Yr* genes, which encode immune receptors or defense-related proteins that directly or indirectly recognize pathogen effectors and initiate early immune responses. *Pst* overcomes host resistance through mutation, recombination, and selection for virulent races that evade recognition by several *Yr* genes (e.g., *Yr5*, *Yr10*, *Yr15*, *Yr17*, *Yr27*, and *Yr48*), which remain effective in many regions but show vulnerability in others against emerging virulent isolates [129,130,131].

Long-term virulence surveys indicate that *Yr5* and *Yr15* retain broad effectiveness against many *Pst* populations, whereas virulence to other *Yr* genes varies across regions and pathogen race structures [129,132]. This highlights rapid pathogen population shifts driven by host-mediated selection. The emergence of virulent lineages and effector variants, including *PstS1*, *PstS2*, *PstS4*, and *PstS7*, along with Avr effectors (e.g., *AvrYrSP*, *AvrYr10*, *AvrYr24*, *AvrYr27*, *AvrYr44*, and *AvrYr76*), enables the pathogen to evade host recognition and suppress early immune responses [60,133,134]. This continuous host-pathogen co-evolutionary arms race underpins the rapid breakdown of race-specific resistance in wheat-growing regions.

### 6.2. Signal Transduction Layer: Disruption of Defense Signaling Networks

Following pathogen recognition, effective resistance depends on activation of interconnected defense signaling pathways, including SA, ROS, MAPK cascades, and transcriptional regulators. *Pst* effectors have been reported to interfere with this signaling layer by targeting key regulatory components, resulting in suppression or delayed activation of host defense pathways. Critical regulators affected include *TaNPR1*, *TaPAD4*, *TaRbohD*, *TaWRKY45*, *TaNAC8*, and *TaMYB30*, which play central roles in immune signal amplification and transcriptional regulation. As a result, SA- and ROS-mediated defense signaling pathways (two central pillars of plant immunity) are significantly impaired [86,135,136]. This disruption of signal transduction represents a key mechanistic step linking effector activity to compromised resistance expression.

### 6.3. Downstream Defense Responses and Climate-Mediated Destabilization

The effectiveness of resistance ultimately depends on downstream defense mechanisms, including ROS accumulation, activation of *PR *↓proteins (*PR1*, *PR2*, and *PR5*), and antioxidant enzyme systems (*TaCAT*, *TaSOD*, and *TaPAL*). The cloned resistance loci of the host, such as *Yr5*/*YrSP*/*Yr7* (*BED-NLRs*) [136], *Yr10* (*NLR*) [137], *Yr15* (*WTK1*; tandem kinase) [138], *Yr18*/*Lr34* (ABC transporter) [139], *Yr36*/*WKS1* (kinase-START) [140], *Yr46*/*Lr67* (hexose transporter) [141], *YrU1* (ank*Yr*in-WRKY-NLR) [142], and *YrNAM* (NAM/ZnF-BED) [143], demonstrate the involvement of multilayered defense mechanisms. Pathogens counter these loci through a wide range of effectors that strategically interfere with their functions. For instance, *Pst-12806* targets wheat *TaISP* in the chloroplast to inhibit photosynthesis-linked ROS production [136], *Pst-9302* interacts with *TaVDAC1* to suppress ROS signaling and programmed cell death (PCD) [143], and Hasp98 interferes with *TaMAPK4* kinase activity to delay MAPK cascade signaling [144]. Additional effectors further disrupt SA biosynthesis and immune regulation [145,146,147,148,149].

In contrast to race-specific resistance, APR, including HTAP resistance, is generally more durable due to its quantitative nature, although it remains sensitive to environmental conditions. Climate-induced stress significantly amplifies resistance instability. Heat, drought, and humidity shifts reduce stomatal conductance and antioxidant enzyme activities (SOD, POD, CAT) and alter SA, JA, and ET signaling crosstalk, thereby destabilizing NLR- and APR-mediated resistance mechanisms.

Fu, Uauy, Distelfeld, Blechl, Epstein, Chen, Sela, Fahima and Dubcovsky [140] reported that *Yr36* (*WKS1*), which confers high-temperature adult-plant resistance through kinase-START signaling, loses efficacy above 30 °C. Similarly, drought and heat stress reduce SA-dependent signaling and increase abscisic acid (ABA) levels, facilitating *Pst* establishment [149,150,151,152]. Transcriptomic studies by Cheng, Luan, Meng, Sun, Tao and Zhao [152] and Guo, et al. [153] reveal suppression of *TaWRKY70*, *TaNPR1*, and *TaRbohB*, along with induction of heat shock proteins (*TaHSP70* and *TaHSP90*) under combined heat and rust stress. eCO_2_ further modifies host-pathogen interactions by increasing canopy density and nighttime humidity, promoting multiple infection cycles within a single season [154]. High CO_2_ also alters the C:N balance and down-regulates defense-associated pathways, including PAL-mediated phenolic production and *PR5* expression [155,156].

**Figure 5:**
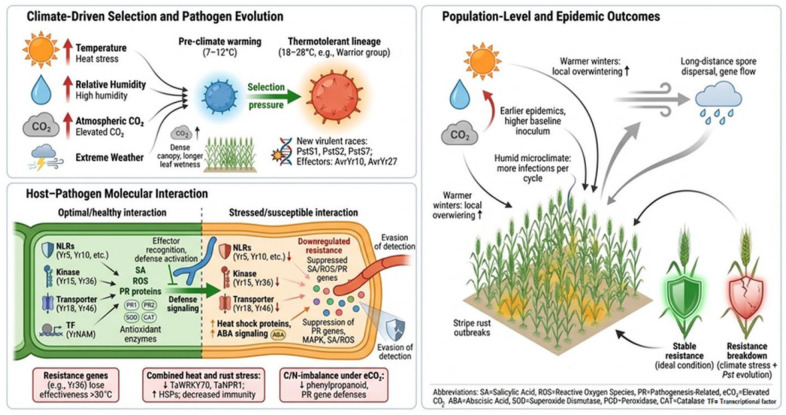
Temperature-driven adaptation and climate-assisted resistance breakdown in wheat stripe rust. This schematic summarizes how climate factors (temperature, humidity, elevated CO_2_, and extreme weather) drive pathogen evolution, alter host molecular defenses, reshape epidemic dynamics of *Pst*, favoring thermotolerant and more virulent lineages. These processes highlight the dynamic Genotype × Environment × Pathogen interaction that influences resistance durability under climate change. ↑ shows an elevated levels of environmental factors.

Consequently, previously effective resistance genes (e.g., *Yr10*, *Yr15*, *Yr36*, *Yr18*, and *Yr46*) may exhibit reduced stability under combined climatic and evolutionary pressures, enabling the emergence of more virulent *Pst* races capable of infecting previously resistant wheat cultivars. How key climate drivers create the selection pressure for *Pst* is illustrated in Figure 5 based on published studies on pathogen evolutionary dynamics and stripe rust epidemiology under changing climates. These findings indicate that resistance breakdown arises from the integrated effects of pathogen evolution, resistance architecture, and climate-driven physiological stress, rather than from the failure of individual genes alone.

## 7. Breeding for Durable and Climate-Resilient Resistance

Breeding remains the most reliable and primary sustainable approach for managing the *Pst* disease in the current era of climate change. From a breeding perspective, resistance strategies can be broadly categorized into race-specific (ASR), adult-plant resistance (APR/HTAP), and their combined deployment for durability under climate stress. Now breeding for stripe rust cannot rely on a single race-specific gene due to the rapid emergence of new *Pst* races. Many studies revealed that temperature fluctuations, eCO_2_, and drought stress suppress the expression of key defense regulators (*TaNPR1*, *TaWRKY70*, and *TaRbohB*), which in turn decrease the efficacy of classical resistance genes like *Yr10*, *Yr15*, and *Yr36* [157,158,159,160]. The recent detection of virulent *Pst* isolates overcoming *Yr15* in the UK in 2025 highlights the vulnerability of even highly durable resistance under combined climatic and evolutionary pressures, although *Yr*15 remained broadly effective across major agroecological zones [77]. Because climate stress simultaneously favors pathogen aggressiveness and weakens host defense, modern breeding programs must shift toward climate-resilient varieties that are more durable and thermotolerant with multi-gene resistance.

Modern resistance breeding should prioritize the combination of all-stage resistance (ASR) with adult-plant, quantitative resistance, some of which is influenced by temperature, to ensure durable performance across environments. This integrated strategy is a central principle of CIMMYT-led wheat improvement programs aimed at long-term stripe rust control. Up to now, more than 80 *Yr* genes and over 300 QTLs associated with stripe rust resistance have been identified in wheat [161,162,163]. The number of reported resistance loci continues to increase with advances in genomic tools, genome-wide association studies (GWAS), and high-throughput mapping approaches [132]. Many of the early breeding programs around the world relied on several race-specific ASR genes (e.g., *Yr1*, *Yr2*, *Yr3*, *Yr4*, *Yr6*, *Yr7*, *Yr9*, *Yr10*, *Yr17*, *Yr24*/*26*, and *Yr27*). These genes provide strong early-stage protection to the plant from infection; however, due to strong selection pressure, new pathotypes appear rapidly that overcome these genes, making them often short-lived [161,164]. In contrast, APR genes provide partial but long-lasting resistance. This makes them more durable and valuable for breeding programs aiming for long-term protection [165,166]. The characterized pleiotropic APR genes include *Yr18*/*Lr34*/*Sr57*/*Pm38*, *Yr29*/*Lr46*/*Sr58*/*Pm39*, *Yr30*/*Sr2*/*Lr27*/*Pm70*, and *Yr46*/*Lr67*/*Sr55*/*Pm46*, which have been widely deployed in international breeding programs, including CIMMYT germplasm and confer partial, slow-rusting resistance with reduced selection pressure on the pathogen population [167]. This partial and quantitative nature of APR limits the rapid emergence of virulent races, making it more sustainable over time. One important form of APR is HTAP resistance. This type of resistance becomes stronger as the plant grows older and with the rise in temperatures. HTAP resistance represents a specialized form of APR that becomes more effective at later growth stages and under elevated temperatures [168]. Wheat plants expressing HTAP resistance are often vulnerable to stripe rust at the seedling stage; however, the level of resistance may increase with the growth progression and attain maximum expression at the adult stage when temperatures become higher [169,170]. This temperature-dependent expression links resistance performance directly to environmental conditions.

Therefore, the durability of resistance is closely linked to the type of resistance deployed and the evolutionary pressure exerted on pathogen populations. ASR genes such as *Yr5*, *Yr9*, *Yr17*, and *Yr27* provide strong seedling resistance but are highly race-specific, and their resistance is lost at all growth stages once virulent *Pst* races emerge, as shown in Table 4 [171]. Conversely, APR and HTAP genes, including *Yr18*/*Lr34*/*Sr57*/*Pm38* (classic pleiotropic APR gene used globally), *Yr30*/*Sr2*, *Yr29*/*Lr46*/*Sr58*/*Pm39* (widely used slow-rusting gene), *Yr36*, *Yr52*, *Yr62*, *Yr46*/*Lr67*/*Sr55*/*Pm46* and *Yr78*, are non-race-specific resistance and give durable resistance across diverse temperature regimes [132,167,172,173]. Their broad-spectrum, non-race-specific activity makes them important components of climate-smart breeding programs. The pyramiding of multiple APR and HTAP genes, a strategy widely implemented in CIMMYT germplasm, has proven effective in stabilizing resistance performance over time and across environments, thereby aligning resistance breeding with the demands imposed by climate variability and the rapid evolutionary capacity of stripe rust populations. This highlights that durable resistance is not determined by individual genes but by the strategic integration of multiple resistance types under evolving pathogen pressures and changing climatic conditions.

## 8. Genomic and Molecular Approaches for Climate-Resilient Stripe Rust Resistance

The rapid advances in genomics, transcriptomics, and computational breeding have transformed how researchers develop durable resistance to stripe rust in wheat. Modern molecular breeding tools now integrate marker-assisted selection (MAS), genome-wide association studies (GWAS), and genomic selection (GS) to accelerate the introgression and prediction of climate-resilient genotypes. MAS has been particularly effective in pyramiding complementary *Yr* genes such as *Yr5*, *Yr15*, *Yr18* (*Lr34*/*Sr57*), and *Yr36* (*WKS1*) into elite varieties and thus enhanced not only temperature stability but also resistance durability [25,183]. Recent GWAS and multi-environment QTL mapping have increased the number of recognized resistance loci far beyond the classical *Yr* genes [184]. Newly characterized loci such as *YrSP*, *YrZH84*, *Yr90*, *Yr82*, and *YrSDG1* have demonstrated resistance that remains effective across wide temperature ranges and multiple *Pst* pathotypes [185,186,187]. Integration of environmental covariates into genomic prediction (GP) models has enabled the identification of genotypes with both high resistance and adaptive performance under simulated warming scenarios [188,189]. These enviro-genomic models are particularly valuable for breeders to anticipate how lines will perform under warm climates before they are tested in the field.

At the molecular level, multi-omics and transcriptome profiling have revealed how wheat crosstalk between biotic and abiotic stress signaling under *Pst* infection [190]. Transcriptomic and functional analyses indicate that WRKY, NAC, and MYB transcription factors act as central regulators of wheat defense under combined rust and heat stress. Overexpression of *TaWRKY45* increases *PR1*/*PR2* expression and broad disease resistance [191], while *TaWRKY70* is positively involved in high-temperature seedling resistance and associated with activation of SA/ET defense signaling [159]. Similarly, *TaNAC69* up-regulates stress-responsive defense genes under abiotic stress [192], and *TaMYB391* regulates PR genes and HR-associated responses against stripe rust [193]. These transcriptional factors, together, coordinate systemic acquired resistance (SAR) through activation of *TaNPR1*, *TaPR1*, and *TaPR5* in response to rust infection.

Heat-induced stress also affects the phenylpropanoid pathway by suppressing biosynthetic genes such as *TaPAL*, *TaC4H*, and *TaCOMT*. This led to reduced lignification, and weakened antioxidant defenses [194,195]. Similarly, *TaRbohB*-mediated ROS production and TaMAPK3/6 cascades are modulated to balance oxidative bursts with cell viability [196]. Heat shock proteins (HSPs) such as *TaHSP70* and *TaHSP90* play a dual role, stabilizing immune receptors (NLRs) and facilitating proper folding of resistance-related kinases under temperature stress [147,197,198]. Co-expression of *TaHSP90* with *TaRGA4-TaRGA5* receptor complexes has been shown to maintain effector recognition under heat stress and reduce breakdown of seedling resistances (ASR genes) [199,200]. These findings are being exploited through QTL mapping and co-expression network analyses to identify stable regulatory hubs for climate-adaptive resistance.

CRISPR-based editing has also revolutionized the breeding of stripe rust-resistant wheat by precisely modifying susceptibility and defense regulatory genes. Recent breakthroughs include knockout of the susceptible gene, such as *TaPsIPK1* [201], and targeted editing of *TaSTP14* [202], which confers broad-spectrum rust resistance without a yield penalty. The integration of multiplex CRISPR editing with genomic prediction models that incorporate genotype × temperature now provides a powerful framework for breeding wheat with durable, multi-gene, climate-adaptive stripe rust resistance.

**Figure 6:**
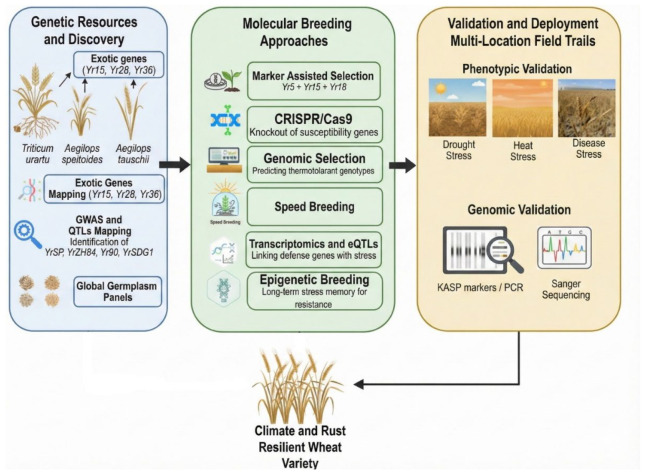
Strategic roadmap for developing climate-resilient and stripe rust-resistant wheat through integrated omics and advanced breeding approaches. This framework illustrates the translational pathway from the identification of novel resistance alleles in wild progenitors (such as *Aegilops tauschii*) using genome-wide association studies (GWAS) and QTL mapping, to their precise introgression into elite cultivars through advanced molecular tools, including CRISPR/Cas9 and speed breeding. The pipeline further incorporates multi-location phenotypic screening and genomic validation (e.g., KASP markers) to ensure the development and deployment of wheat varieties capable of withstanding complex biotic and abiotic stresses.

Epigenetic modulation has also been implicated in plant responses to biotic stress, although its application in breeding remains an emerging area. Recent methylation studies indicate that *Pst* infection under high temperature alters DNA methylation at *TaPR1* and *TaPAL* promoters, suppressing defense responses [176]. These findings suggest a potential regulatory role of epigenetic modifications in host–pathogen interactions. However, breeding for stable epigenetic marks associated with rust resistance is still largely exploratory and requires further validation under field conditions. Conversely, several studies have shown that wheat possesses temperature-responsive DNA demethylases and that promoter methylation in wheat can change dynamically under heat and cold stress. While genes such as *TaWRKY45* and *TaNPR1* are well-established regulators of plant defense, the role of their epigenetic regulation in conferring durable stripe rust resistance is not yet fully established [191,203]. These findings, taken together, suggest that epigenetic regulation may contribute to transcriptional plasticity under fluctuating temperatures, but its direct application in breeding programs remains limited at present. Integration of genomic, transcriptomic, and epigenomic datasets now offers a holistic framework for developing wheat cultivars resilient against rust and climate stress. HTAP genes like *Yr18*, *Yr36*, and *Yr78*, coupled with genomic prediction models accounting for temperature-by-genotype interactions, contribute to the long-term sustainability to breeding programs (Table 5). The convergence of multi-omics-assisted breeding and AI-driven predictive selection thus represents the next frontier in ensuring global wheat security under a warming climate. The integrated framework combining allele discovery, molecular breeding, and multi-environment validation is illustrated in Figure 6.

## 9. Artificial Intelligence and Machine Learning in Stripe Rust Prediction and Management

In recent years, stripe rust has become more unpredictable because climate conditions are shifting more quickly than in the past. These changes have pushed researchers to look beyond traditional forecasting tools and explore data-driven approaches. Many historical disease forecasting models relied on empirical temperature and humidity thresholds. These models were valuable in the past but sometimes struggle to accommodate the complex interactions between environment, host physiology, and pathogen that are now emerging under changing climates [98,213]. Some models still perform well in some regions, but they should be updated and validated using the most recent and validated data and advanced approaches.

Currently, Artificial Intelligence and Machine Learning tools are increasingly being used to support understanding, prediction, and management of stripe rust [214,215]. ML algorithms such as Random Forests [216] and Support Vector Machines [217] improve predictive accuracy by learning from multi-dimensional datasets comprising historical disease records, high-resolution weather data, canopy temperature, relative humidity, spore trapping, and crop growth stages (Table 6). Such models can be used to create early warning risk maps, predict epidemic onset, and identify the environmental windows that may be conducive to a rapid infection. For instance, Ruan, Dong, Huang, Huang, Ye, Ma, Guo and Sun [216] used time-series Sentinel-2 satellite imagery to compute vegetation indices (VIs) sensitive to disease stress and combined these with meteorological variables such as temperature, rainfall, and relative humidity. In a study dealing with this approach, a Random Forest classifier using phenology-based VIs (indices adjusted for the particular growth stage of wheat) plus meteorological variables achieved an overall accuracy of around 88–89% [216]. Similarly, a range of machine learning models and their predictive performance for stripe rust occurrence and disease severity have been reported, integrating satellite imagery with climatic and crop-related variables, as summarized in Table 6.

**Figure 7:**
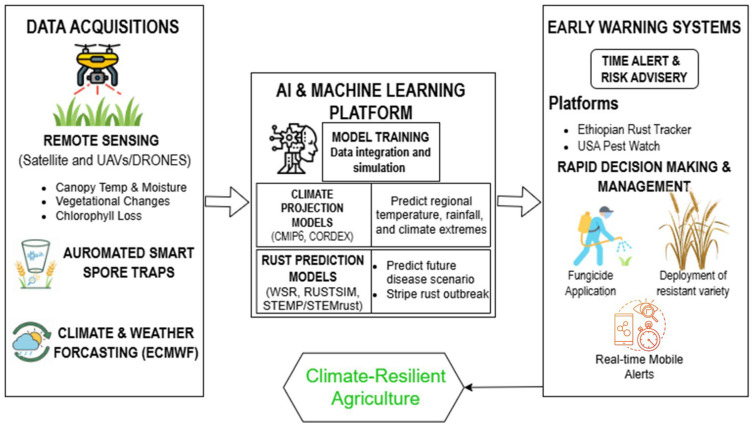
This diagram illustrates an integrated workflow combining multi-source data acquisition (remote sensing, spore traps, and weather forecasting) with an AI and Machine Learning platform. The system utilizes climate projection and rust prediction models to generate early warning alerts, facilitating rapid decision-making and disease management strategies.

This requires a layered and integrated optimization of AI for stripe rust under climate change. First, a coordinated network is required that integrates spore traps, measured airborne inoculum, remote sensing by satellite or UAV, and leaf-level ground imaging to produce multi-modal data, as depicted in Figure 7. Second, ML algorithms can transform this information into a risk assessment that identifies the likelihood of infection. Third, translation of these risks into actionable and eco-friendly advisories that help farmers to decide fungicide application, cultivar choice and how to adjust agronomic and management practices during high-risk periods. AI can also play a supporting role in resistant breeding. Tools such as genomics and GWAS data help breeders to identify resistance loci. Although the integration of climate base ML forecasting with genomic breeding pipelines is still experimental, it offers a potential path toward selecting for those that not only carry resistant genes but also maintain their performance under warmer temperatures and variable moisture conditions. Therefore, the integration of AI, ML, and multi-source environmental and genomic data provides a transformative approach to predict and manage stripe rust. The current application of AI and machine learning approaches for stripe rust prediction is not without limitations that must be acknowledged. Model performance and accuracy remain highly dependent on the availability, quality and temporal continuity of input data, which are uneven across regions. These limitations arise particularly in low- and middle-income wheat-growing areas. Many models are trained using local datasets and may not work well when applied to different climates, cropping systems and rust populations. In many wheat-growing areas, access to real-time weather data, remote sensing products and digital decision support tools is still limited. Bridging this gap will require better disease surveillance, long-term data collection, and closer integration of AI tools with field observations and extension services.

## 10. Integrated Disease Management Under Climate Change

Genetic resistance alone may not be enough under the rapidly changing climate. Genetic resistance determines the baseline level of disease suppression, but its durability is strongly influenced by agronomic decisions such as sowing date, irrigation scheduling, and nitrogen management, which shape crop microclimate and disease pressure. Climate change has already reshaped the behavior of *Pst* by allowing it to survive in warmer winters, spread faster through unpredictable wind patterns, and evolve new virulent races capable of breaking resistance genes. Therefore, there should be an integrative management of this disease through the combination of cultural practices, healthy soils, biological tools, smart chemical use, and continuous monitoring. The foundation of stripe rust management still begins with the right variety. However, even the best variety cannot stand alone if the primary inoculum is high and environmental conditions are favorable. This is where cultural and agronomic practices form the first line of defense by reducing the availability of primary inoculum in the field [222].

Adjusting sowing dates by one to two weeks can help farmers avoid the seasonal window when spores are airborne at their highest rate. Management of crop residues and crop rotation with nonhost plants greatly reduces the chance for *Pst* to survive the off-season and break its disease cycle. Balanced fertilization, with controlled nitrogen use, along with avoidance of heavy irrigation that prolongs leaf wetness, also reduces the rust infection. In contrast, excessive nitrogen fertilization can extend the period of canopy susceptibility and increase leaf wetness, thereby intensifying rust development and placing greater selection pressure on resistance genes [223].

Chemical control remains a key component of wheat stripe rust management, particularly under high disease pressure, with demethylation inhibitor (DMI) and quinone outside inhibitor (QoI) fungicides widely used for effective disease suppression and yield protection [49,224]. However, increasing reliance on fungicides has raised concerns regarding resistance development. Widespread resistance in *Pst* is not yet fully established, but several studies have reported reduced sensitivity to DMI fungicides associated with mutations and overexpression of the *CYP51* gene, indicating an emerging risk [224]. Recent evidence suggests that such sensitivity shifts may already be occurring at regional scales, highlighting fungicide resistance as a growing global concern. Under climate change scenarios, characterized by increased temperature variability and the emergence of more aggressive pathogen races, stripe rust epidemics are expected to become more frequent, leading to increased fungicide applications and stronger selection pressure for resistance [225,226]. In this context, contemporary management increasingly relies on monitoring, early detection, and weather-based forecasting systems, which enable targeted and site-specific fungicide applications. Such approaches improve timing, reduce unnecessary use, and help delay resistance development while minimizing environmental impacts [227]. Therefore, integrating chemical control with resistance management strategies, including rotation of modes of action, optimized application timing, and combination with host resistance and biological approaches, is essential for sustaining fungicide efficacy under changing climatic conditions.

Different BCAs, such as *Bacillus subtilis*, *Trichoderma harzianum*, *Pseudomonas fluorescens*, and arbuscular mycorrhizal fungi (AMF), have shown their potential through various studies and field trials in suppressing *Pst* by competition, antibiosis, and induction of resistance in a host system [228,229,230,231]. Quantitative evidence from controlled and field-based studies indicates that BCAs can reduce stripe rust severity by approximately 30–70%, depending on the microbial strain, environmental conditions, and application timing [232,233,234]. First instance, Li, et al. [235] found that the endophytic bacterium *Bacillus subtilis* strain E1R-j significantly suppressed wheat stripe rust, reducing disease severity by up to 87.7% under controlled greenhouse conditions, along with significant inhibition of urediniospore germination. More recent studies further support the high efficacy of bacterial antagonists. For example, *Paenibacillus polymyxa* strain XD29-G1 exhibited control efficiencies of 55.97% (culture solution), 61.19%, and up to 65.84% under protective treatment conditions, with consistent suppression above 60% in pot experiments [232]. Similarly, other endophytic bacteria such as *Paenibacillus xylanexedens* and *Bacillus megaterium* have been reported to reduce stripe rust severity by 61.11% and 65.16%, respectively [236]. Their performance is highly dependent on environmental factors such as temperature, humidity, and UV exposure, which influence microbial survival and activity on the leaf surface [230]. In addition to these BCAs, biofungicides derived from such microbial antagonists are increasingly being explored as an effective and sustainable component of stripe rust management. Commercial biofungicide formulations based on *Bacillus subtilis* (e.g., strain QST713) also demonstrate measurable field efficacy, achieving up to 60% disease control under moderate disease pressure, although performance may decline to <30% under high disease pressure conditions. These biofungicides function through multiple mechanisms, including the production of antifungal metabolites, niche exclusion, and the activation of plant systemic resistance pathways [236]. Despite these limitations, BCAs play an important complementary role in integrated disease management by reducing fungicide dependence, lowering selection pressure for resistance development, and contributing to environmentally sustainable disease control strategies. Therefore, integrating BCAs with resistant cultivars and climate-informed fungicide applications provides a more resilient and adaptive strategy for managing stripe rust under changing climatic conditions.

## 11. Conclusions and Future Perspectives

Stripe rust will continue to be one of the most dynamic and unpredictable threats to wheat production worldwide. This disease is becoming more pervasive under climate change and causes significant yield losses. Conventional control measures are often insufficient on their own because the *Pst* can rapidly spread, with high mutation rates, resulting in the emergence of new races and adaptability to a changing environment. In the coming decades, continued global warming is likely to favor the expansion of thermotolerant *Pst* lineages into previously cooler regions, while also increasing epidemic frequency and extending the infection window within cropping seasons. Consequently, future *Pst* populations are expected to exhibit enhanced thermal adaptation, broader virulence spectra, and increased epidemic potential. A more proactive and predictive management approach will therefore be essential to address this evolving threat. Though there are different yellow rust management methods being practiced, the deployment of resistant cultivars remains the cornerstone of integrated control of yellow rust. However, the durability of resistance is frequently compromised following the deployment of new resistance genes. Future *Pst* populations are expected to increasingly overcome race-specific resistance due to continuous mutation-driven evolution, somatic recombination, and large-scale dispersal of aggressive lineages. Future breeding programs must therefore prioritize gene pyramiding strategies that combine major R genes with APR and heat-stable QTLs. Advances in molecular tools such as genomic selection and CRISPR-based gene editing will accelerate the development of cultivars that are not only high-yielding but also capable of withstanding shifting rust races and fluctuating climate conditions. Enhancement of regional rust surveillance networks, such as the Global Rust Initiative (GRI), provides support for rapid identification of new races and guides breeders in selecting effective resistance sources. Early detection and management of stripe rust is another solution for managing this disease. However, this requires the integration of advanced technological tools with traditional field-based monitoring. Future research should focus on developing high-resolution pathogen surveillance systems that incorporate remote sensing, UAV imagery, and machine-learning-based early warning models. Such technologies can identify an infection before the appearance of visual symptoms. This enables timely intervention to reduce disease spread and crop losses. Climate-smart integrated pest management (IPM) strategies are essential, and future approaches will likely rely on predictive disease modeling, real-time surveillance, and adaptive management practices to respond to rapidly evolving pathogen populations. Future research should explore optimized sowing dates, precision irrigation, and nutrient management practices that reduce crop stress and lower susceptibility to rust infection. Integrating real-time DSS, mobile-based advisory platforms, and automated weather-rust models will facilitate responsive and efficient IPM adoption. In future agro-ecosystems, the integration of predictive epidemiological models with climate projections will be critical for anticipating outbreak risks and guiding timely interventions. The integration of BCAs, biofungicides, and resistant cultivars represents a key component of sustainable stripe rust management, reducing reliance on chemical control while minimizing environmental impacts, particularly under changing climatic conditions. The future management of stripe rust will require a multidisciplinary and adaptive approach that integrates advances in genomics, epidemiology, climate science, and digital agriculture. The use of predictive epidemiological models linked with climate projections will be essential for anticipating outbreak risks and enabling proactive disease management. Continuous monitoring, innovation, and integration of technologies will be critical to safeguarding global wheat production against this rapidly evolving pathogen.

## Figures and Tables

**Table 4:** Classes of *Yr* genes and their temperature response profile against *Pst*.

Resistance Type	Gene	Temperature Response	Durability/Climate Relevance	Reference
ASR (race-specific)	*Yr5*, *Yr10*, *Yr15*, *Yr17*, *Yr27*	More effective at the lower temperature Many ASR genes lose efficacy as the temperature rises, usually >18 °C	High efficacy at cool temperatures; rapidly overcome under warming	[174]
APR/HTAP	*Yr18*/*Lr34*/*Sr57*	Enhanced at 20–25 °C	Durable, non-race-specific resistance with a broad spectrum	[172,175]
APR/HTAP	*Yr36* (*WKS1*)	Induced at ≥23 °C	Confers thermotolerant defense via the kinase-START protein	[140]
APR/HTAP	*Yr52*, *Yr59*, *Yr62*, *Yr78*, *Yr79*	Stable at 22–26 °C	High resistance under elevated temperature	[176,177,178,179]
APR (newly mapped)	*Yr81*, *Yr82*, *Yr83*	Not temperature-sensitive	Promising for multi-pathotype resistance	[180,181,182]

**Table 5:** Key modern breeding and molecular approaches for climate-robust stripe rust resistance in wheat.

Approach	Key Examples	Outcome	References
MAS	Pyramiding *Yr5 + Yr15 + Yr18*	Durable multi-gene protection	[204]
GWAS/QTL mapping	Identification of *YrSP*, *YrZH84*, *Yr90*, *YrSDG1*	Discovery of climate-robust loci	[205,206]
Genomic Selection (GS)	Multi-environment GS with climatic data	Predicting thermotolerant genotypes	[207]
Transcriptomics and eQTLs	*TaHSP70*, *TaWRKY45*, *TaNPR1* under heat + *Pst*	Linking defense regulation with stress	[159,208,209]
Epigenetic Breeding	DNA methylation in *TaPAL*, *TaPR1*	Long-term stress memory for resistance	[136,210]
CRISPR/Cas9 Editing	Targeting *TaSTP13*, *TaPsIPK1*	Disruption of susceptibility genes	[201,211]
Multi-omics Integration	Genomic + Proteomic + Metabolomic networks	Systems-level identification of climate-resilient regulators	[210,212]

**Table 6:** This table summarizes a selection of representative AI and machine learning models developed to predict or detect stripe rust, as reported in the literature.

Model/Algorithm	Dataset and Inputs	Sensor/Data Source	Performance Metrics	References
Random Forest	97 field plots phenological VIs + meteorological features	Sentinel 2 time series + ground meteorological stations	Accuracy = 88.7%	[216]
Support Vector Machine (SVM)	58 field survey plots, time-series features	Sentinel 2 (16 VIs, optimized via Sequential Forward Selection)	Accuracy range = 65.5% to 86.2%	[217]
Discriminant Partial Least Squares (DPLS) and SVM	Canopy spectral data labeled by *Pst* quantity (duplex PCR)	Hyperspectral (325–1075 nm)	Recognition accuracy = Nearly 75–80%	[218]
Image-processing + ML (Random Forest, etc.)	1827 training, 457 test images	Leaf/canopy images (machine vision)		[219]
Random Forest after SLIC superpixel + segmentation	2284 image patches (1827 training, 457 test) from leaf images	Machine-vision (RGB leaf photos)	Perceptual lesion area loss = 0.064	[220]
RF, XGBoost, SVM (feature set selected via Geographical Detectors)	94 survey points	Sentinel 2 time series + meteorological + spatial features	Best (GD-RF): Accuracy = 87.2%, Kappa = 0.743	[221]
Environmental response regression modeling (multiple regression)	5 years (2013–2017) severity data on 3 wheat varieties + validation for 2018–2019	Meteorological data: max/min temp, RH, rainfall, wind speed	Explained variability is almost 89%	[14]
Rule-based weather-threshold model	Data from 98 + 99 fields across 9 sites (2018–2019)	Weather-station data (RH, rainfall, temperature)	Probability of Detection ≥ 0.92	[119]

## Data Availability

All information analyzed and discussed is derived from previously published studies and is fully provided within the manuscript.

## Abbreviations

The following abbreviations are used in this manuscript:

ABA	Abscisic Acid
AI	Artificial Intelligence
AMF	Arbuscular Mycorrhizal Fungi
APR	Adult-Plant Resistance
ASR	All-Stage Resistance
BCA	Biological Control Agent
BCAs	Biological Control Agents
CAT	Catalase
CDL	Cereal Disease Laboratory
CO_2_	Carbon Dioxide
CSI	Critical Success Index
DPLS	Discriminant Partial Least Squares
CIMMYT	International Maize and Wheat Improvement Center
eCO_2_	Elevated Carbon Dioxide
ET	Ethylene
FACE	Free-Air CO_2_ Enrichment
IPM	Integrated Pest Management
GD	Geographical Detectors
GP	Genomic Prediction
GRI	Global Rust Initiative
GRRC	Global Rust Reference Center
GS	Genomic Selection
GWAS	Genome-Wide Association Study
HSPs	Heat Shock Proteins
HTAP	High-Temperature Adult-Plant Resistance
JA	Jasmonic Acid
LAI	Leaf Area Index
MAPK	Mitogen-Activated Protein Kinase
MAS	Marker-Assisted Selection
ML	Machine Learning
MYB	MYB Transcription Factor Family
NAC	NAM, ATAF, and CUC Transcription Factor Family
NLR	Nucleotide-Binding Leucine-Rich Repeat
PAL	Phenylalanine Ammonia-Lyase
PCD	Programmed Cell Death
POD	Peroxidase
PR	Pathogenesis-Related Protein
*Pst*	*Puccinia striiformis* f. sp. *tritici*
QTL	Quantitative Trait Locus
RF	Random Forest
RH	Relative Humidity
ROS	Reactive Oxygen Species
SA	Salicylic Acid
SAR	Systemic Acquired Resistance
SOD	Superoxide Dismutase
SVM	Support Vector Machine
VIs	Vegetation Indices
WRKY	WRKY Transcription Factor Family
